# Regulation of gene expression by manipulating transcriptional repressor activity using a novel CoSRI technology

**DOI:** 10.1111/pbi.12683

**Published:** 2017-03-10

**Authors:** Yue Xu, Song Feng Li, Roger W. Parish

**Affiliations:** ^1^ Department of Animal, Plant and Soil Sciences La Trobe University AgriBio – Centre for AgriBioscience Melbourne Vic Australia

**Keywords:** transcriptional repressor, Conserved Sequence‐guided Repressor Inhibition (CoSRI), *MYB80*, *WUSCHEL*, *TOPLESS*, hybrid seed production

## Abstract

Targeted gene manipulation is a central strategy for studying gene function and identifying related biological processes. However, a methodology for manipulating the regulatory motifs of transcription factors is lacking as these factors commonly possess multiple motifs (e.g. repression and activation motifs) which collaborate with each other to regulate multiple biological processes. We describe a novel approach designated conserved sequence‐guided repressor inhibition (CoSRI) that can specifically reduce or abolish the repressive activities of transcription factors *in vivo*. The technology was evaluated using the chimeric MYB80‐EAR transcription factor and subsequently the endogenous WUS transcription factor. The technology was employed to develop a reversible male sterility system applicable to hybrid seed production. In order to determine the capacity of the technology to regulate the activity of endogenous transcription factors, the WUS repressor was chosen. The WUS repression motif could be inhibited *in vivo* and the transformed plants exhibited the *wus‐1* phenotype. Consequently, the technology can be used to manipulate the activities of transcriptional repressor motifs regulating beneficial traits in crop plants and other eukaryotic organisms.

## Introduction

A mechanism of active gene repression shared by all eukaryotes involves the recruitment of co‐repressors and chromatin remodelling factors (e.g. histone deacetylases) by the repression motifs of transcription factors (Krogan and Long, 2009). Histone deacetylation occurs at the regulatory region of the targeted gene and transcription is repressed, that is, a repressed chromatin state results. In Arabidopsis, TOPLESS (TPL) and four TPL‐related (TPR) proteins act as general co‐repressors (Liu and Karmarkar, 2008). The TPL/TPR co‐repressors interact directly with a wide range of transcription factors via their repression motifs (e.g. EAR motifs, WUS box) and recruit a histone deacetylase (HDAs) complex to control diverse hormone signalling and developmental pathways (Causier *et al*., 2012; Jiang *et al*., 2013; Oh *et al*., 2014; Pauwels *et al*., 2010; Szemenyei *et al*., 2008). The N‐terminal domains (amino acids 1–209) of the TPL/TPR co‐repressor bind with the repression motifs of the transcription factors and form dimers and tetramers (Ke *et al*., 2015; Long *et al*., 2006). The N‐terminal domains contain the conserved LisH and CTLH motifs (Long *et al*., 2006). Adjacent to the N‐terminal domains are the proline‐rich motifs. WD‐repeat domains at the C‐termini are required to mediate protein–protein interactions. The domain organization of TPL/TPR and other co‐repressors is similar. Examples of such co‐repressors include LEUNIG (LUG) from Arabidopsis, Tup and SIF from yeast, Groucho (Gro) from Drosophila and human transducin‐like enhancers of split (TLEs) (Chen and Courey, 2000; Liu and Karmarkar, 2008).

Our aim was to develop a system in which co‐repressor‐mediated repression could be reversed and the target gene(s) reactivated. The technology was initially developed to engineer reversible male sterility in plants, a pre‐requisite for hybrid seed production.

The Arabidopsis *MYB80* transcription factor is essential for pollen development and the correct timing of tapetal programmed cell death (PCD) in anthers (Higginson *et al*., 2003; Li *et al*., 1999, 2007; Phan *et al*., 2011; Zhang *et al*., 2007). Premature tapetum degeneration and microspore abortion occurred in the anthers of a *myb80* T‐DNA insertion mutant lacking the final 18 amino acids of MYB80. The mutant is completely male sterile (Li *et al*., 2007). The MYB80 homologues in rice, wheat, canola, cotton and barley exhibit similar expression patterns and strong functional conservation (Li *et al*., 2007; Phan *et al*., 2011; Xu *et al*., 2014). Three genes directly regulated by MYB80 have been identified so far – the A1 aspartic protease UNDEAD, a pectin methylesterase (VANGUARD1) and a glyoxal oxidase (GLOX1) (Phan *et al*., 2011). Advanced tapetal degeneration and premature PCD were observed in the *undead* mutant (Phan *et al*., 2011).

High similarity of the amino acid sequences of MYB80 homologues occurs in the MYB domain (amino acids 1–115), a 44‐amino‐acid region adjacent to the MYB domain (amino acids 125–168), and an 18‐ to 21‐amino‐acid region at the end of the C‐termini. A variable region of 131–139 amino acids is present between the 44‐amino‐acid and the C‐terminal sequences (Li *et al*., 2007; Phan *et al*., 2012; Xu *et al*., 2014). When a 12‐amino‐acid SRDX EAR (LDLDLELRLGFA) motif was fused to the full‐length or C‐terminus truncated MYB80 protein (MYB domain plus the 44‐amino‐acid region), 60%–75% of the transgenic plants were completely male sterile (Li *et al*., 2007; Phan *et al*., 2012). Very few completely male sterile lines were obtained when a 32R EAR (LDLNLELRISPP) sequence from AtMYB32 was used (Xu *et al*., 2014). A means for overcoming the inhibitory effect of MYB80‐EAR on downstream gene expression would provide a novel approach for achieving reversible male sterility in crops.

The *WUSCHEL* (*WUS*) gene encodes a homeodomain transcription factor required for the maintenance and structural integrity of shoot and floral meristems (Laux *et al*., 1996; Mayer *et al*., 1998). *WUS* is expressed during embryogenesis and post‐embryonic development in the organizing centre beneath the stem cells. Distinct regulatory elements in the *WUS* promoter control its expression to define the boundaries of the stem cell niche (Bäurle and Laux, 2005; Mayer *et al*., 1998). *WUS* expression is also detected in the stomium region of anthers from developmental stages 2–11 (Deyhle *et al*., 2007). Mutations in *WUS* result in defective vegetative and inflorescence development. The 7‐day‐old *wus‐1* mutant lacks a shoot meristem and leaf primordia. The mature seedling has disorganized bunches of cauline leaves. Flowers are rarely produced and the produced flowers have reduced numbers of reproductive organs (Laux *et al*., 1996). The *wus‐1* mutant shows a completely sterile phenotype. Its anthers have smaller or malformed locules. In *wus‐1* anthers, neither stomium nor septum cells differentiate or undergo programmed cell death and degenerate. As a consequence, the anther remains intact and fails to dehisce when the pollen grains are matured (Deyhle *et al*., 2007).

Three distinct motifs are located within the WUS C‐terminal region, namely an acidic motif, an EAR‐like motif and a WUS box. The WUS box (TLPLFPMH) is conserved in the WUS homeobox (WOX) protein family in Arabidopsis (Haecker *et al*., 2004; Mayer *et al*., 1998). The conserved WUS C‐terminal domains are involved in protein–protein interactions. Functional analysis of these conserved domains revealed that the WUS protein can act as either a transcriptional repressor or activator, possibly via recruiting different cofactors through the WUS box (Ikeda *et al*., 2009). The mutation in *wus‐1* changes the 5′ exon boundary of intron2 (GG to GA), resulting in a translational stop within the intron after a few codons (Mayer *et al*., 1998). The truncated WUS protein produced in the *wus‐1* mutant would lack the EAR motif and the WUS box.

Using yeast two‐hybrid and GST‐pulldown assays, the conserved C‐terminal domains of WUS have been shown to interact with TOPLESS (TPL) (Kieffer *et al*., 2006). The C‐terminus truncated WUS fused with TPL was able to partially rescue the *wus‐1* mutant (Causier *et al*., 2012), indicating WUS recruits TPL through its C‐terminal region to regulate the expression of downstream genes.

In this study we describe a novel repression motif‐targeted inhibition technology. This technology was employed in Arabidopsis, firstly, to create a reversible male sterility system using MYB80 and, secondly, to manipulate the repressive activity of the WUS homeodomain transcription factor.

## Results

### Designing a CoSRI construct

**Figure 1:**
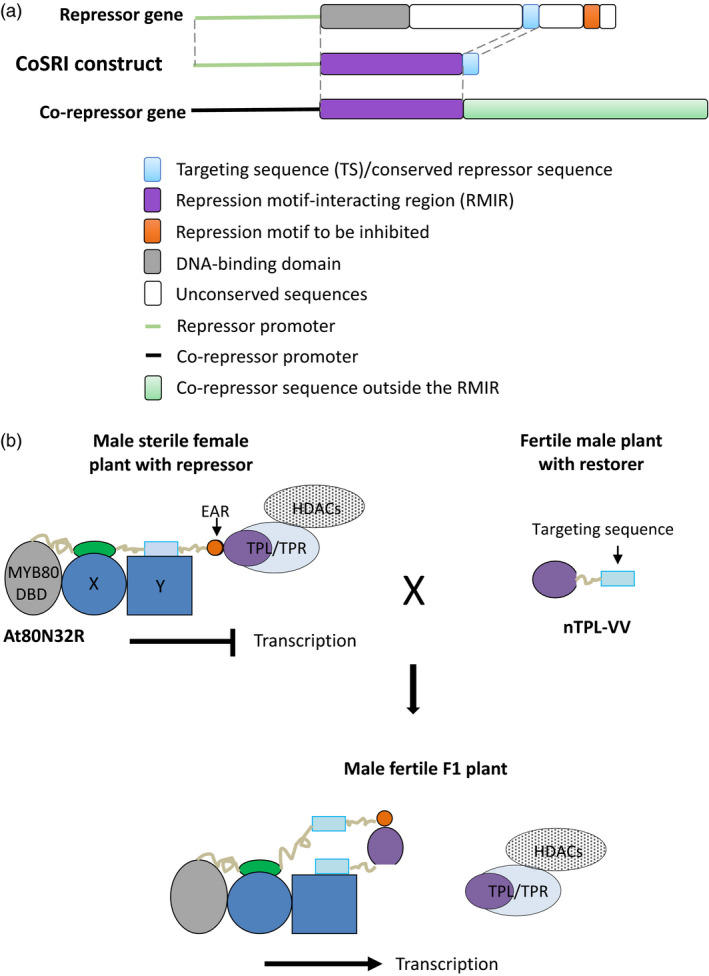
Diagram of the CoSRI construct and the proposed mechanism for CoSRI restorer in the hybrid seed production system. (a) The CoSRI construct contains a repression motif‐interacting region (RMIR) of a co‐repressor and a guide sequence derived from a conserved sequence of the repressor to be inhibited. The construct is driven by the promoter of repressor gene. In this study, the repression motifs, namely the EAR motif in the MYB80‐EAR chimeric transcription factor and the WUS box in the WUS transcription factor, were targeted. The repression motif‐interacting region of the TOPLESS co‐repressor (N‐terminal domain) was used for the CoSRI constructs. (b) The CoSRI restorer (nTPL‐VV) consists of the TPL N‐terminus (TPL_1‐188) fused with a targeting sequence, the 21‐amino‐acid conserved region of the MYB80 C‐terminus. In the fertile male plants, the restorer does not interfere with key EAR repressors in anthers as it possesses low EAR‐binding affinities (see section ‘Discussion’). In the female plants, the MYB80‐EAR (At80N32R) chimeric repressor recruits the TPL/TPR HDACs complex to repress the transcription of MYB80 target genes resulting in male sterility. The two conserved C‐terminal sequences in MYB80 protein are proposed to interact with unknown proteins X and Y, respectively. In the F1 hybrid plants, the targeting sequence guides the restorer to the repressor MYB80‐EAR (i.e. increasing the EAR‐binding affinity) via the proposed protein Y, displacing the TPL/TPR HDACs complex. Consequently, the transcription of the MYB80 target genes is resumed and fertility restored. The proteins in the regulatory complex may occur in multiple copies. MYB80 DBD, MYB80 DNA‐binding domain; TPL/TPR, TOPLESS/TOPLESS‐RELATED co‐repressors; HDACs, histone deacetylases.

In this study we have designated the technology conserved sequence‐guided repressor inhibition (CoSRI). The CoSRI protein consists of the repression motif‐interacting region (RMIR) of a co‐repressor fused with a targeting sequence (TS) derived from the repressor targeted for inhibition (Figure 1a). The construct is driven by the promoter of the repressor gene. The targeting sequence is chosen to be conserved in the orthologues/homologues from different plant species and hence is likely to interact with other factors present in the repressor complex. The CoSRI protein would be recruited into the repressor complex via the targeting sequence to inhibit the interaction of the repression motif with the co‐repressor. A proposed molecular mechanism for the restorer (a CoSRI protein) in a hybrid production system is presented (Figure 1b).

AtMYB80 (Phan *et al*., 2012; Xu *et al*., 2014) and WUS (Kieffer *et al*., 2006) orthologues occur in many plant species. A conserved C‐terminal sequence of 21 amino acids was identified in the AtMYB80 orthologues (Phan *et al*., 2012; Xu *et al*., 2014) and a conserved 30‐amino‐acid sequence found in the WUS orthologues/homologues using the protein BLAST. The two sequences were used as targeting sequences in this study. Sequences conserved in the repressor homologues within the same species may also be employed as targeting sequences although these homologues would be targeted by the CoSRI protein. The repression motif‐interacting region (RMIR) is the minimal length of co‐repressor sequence required to bind the repression motif. The technology can be employed to inhibit the repression motifs interacting with known co‐repressors.

### Male sterility is induced in Arabidopsis by a novel EAR repressor when fused to the MYB80 protein

Strong expression levels of *TPL* were found in wild‐type Arabidopsis anthers from developmental stages 5–9 (Figure S1), indicating the TPL‐histone deacetylase (HDAs) complex is available for recruitment by the MYB80‐EAR chimeric repressor protein. To inhibit the MYB80‐EAR using the CoSRI technology, a more efficient version of the MYB80‐EAR repressor was first constructed. Thus, a modified 12‐amino‐acid peptide GLDLDLNLELRL, designated N32R EAR repressor, was created (see section ‘Materials and Methods’). Its repression capacity was compared with those of the 32R or SRDX EAR motifs.

**Figure 2:**
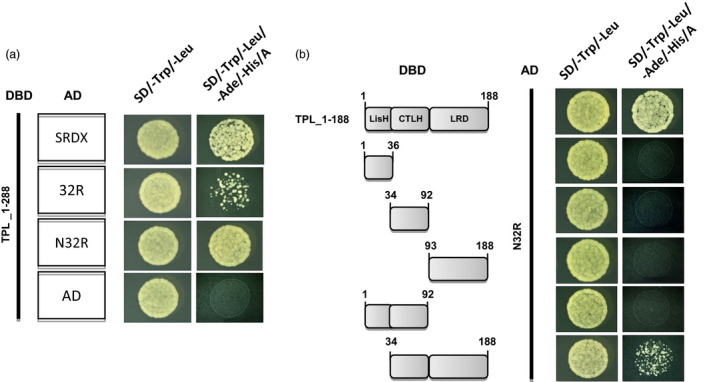
Yeast two‐hybrid analysis of interactions between EAR motifs and the domains in TPL N‐termini. (a) Weak interactions are exhibited between TPL_1‐288‐DBD and 32R‐AD fusion proteins, while strong interactions occur between TPL_1‐288‐DBD and SRDX‐AD or N32R‐AD fusion proteins. (b) Strong interactions occur between TPL_1‐188‐DBD (LisH–CTLH–LRD–DBD) and N32R‐AD fusion proteins. Numbers indicate amino acid residues of TPL N‐terminus. AD, activation domain; DBD, DNA‐binding domain.

The capacity of the TPL N‐terminus (TPL_1‐288, 288 amino acids) to interact with the three EAR motifs SRDX, 32R and N32R was assessed in yeast cells. On quadruple dropout medium, large colonies strongly staining blue were obtained, indicating both the N32R and the SRDX motif interacted strongly with TPL_1‐288. However, interaction between the 32R motif and TPL_1‐288 was relatively weak (Figures 2a; S2). The LisH, CTLH and a leucine‐rich (LRD) domain at the N‐termini are conserved in the TPL/TPR co‐repressor family (Long *et al*., 2006). To determine the region of TPL interacting with the EAR motifs, the truncated N‐terminal domains were tested in yeast. The individual LisH, CTLH, LRD domains failed to interact with N32R. However, a strong interaction took place between the combined LisH–CTLH–LRD peptide (TPL_1‐188, 188 amino acids) and N32R (Figures 2b; S2). The combined N‐terminal domains form a minimal functional region for effectively recruiting various EAR motifs and other repression domains.

**Figure 3:**
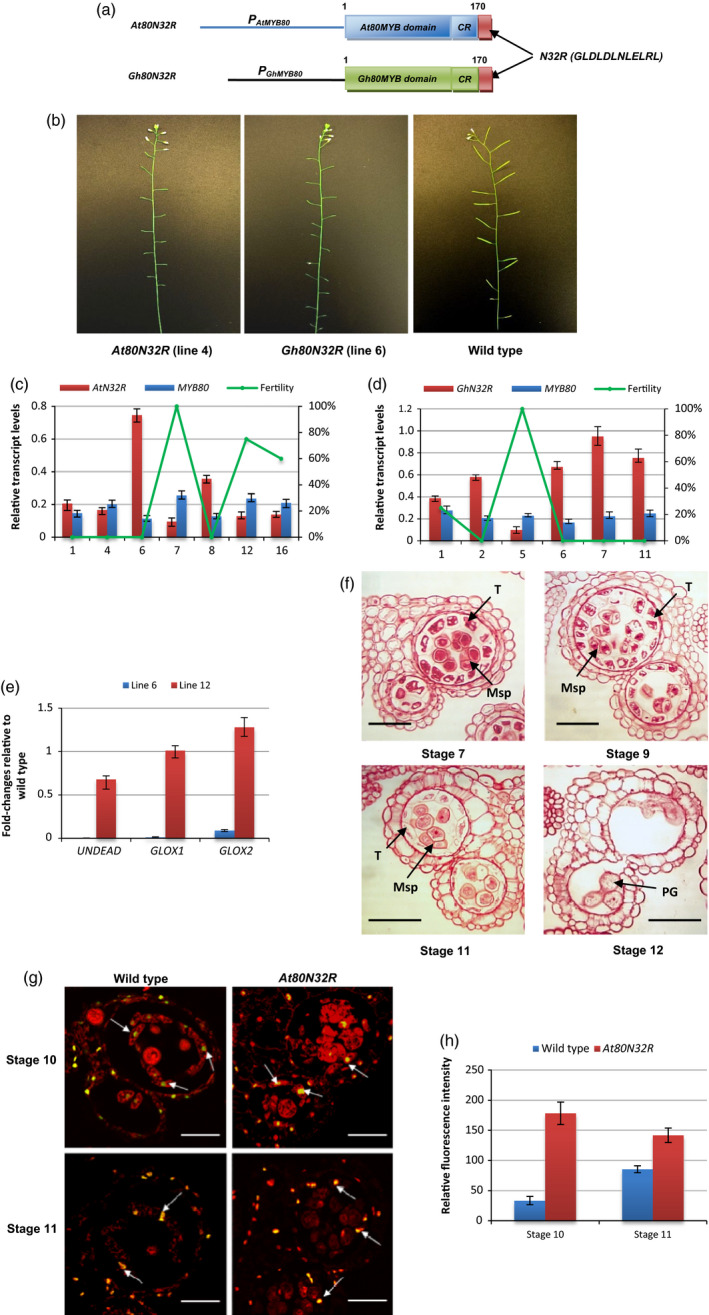
Complete male sterility is induced in Arabidopsis using the N32R repressor motif when fused to MYB80. (a) A schematic diagram (not to scale) of the *At80N32R* and *Gh80N32R* constructs. CR, the *AtMYB80* and *GhMYB80* conserved 44‐amino‐acid region; N32R, a 12‐amino‐acid EAR sequence. (b) The *At80N32R* (line 4) and *Gh80N32R* (line 6) transgenic lines show a completely male sterile phenotype. (c) Plant fertility and relative expression levels of *At80N32R* (*AtN32R*) and endogenous *MYB80* in the selected lines. Expression levels are normalized to *UBQ10*. The Y‐axis on the left represents relative transcript levels and the Y‐axis on the right indicates the fertility (silique elongation). X‐axis represents line numbers. (d) Plant fertility and relative expression levels of *Gh80N32R* (*GhN32R*) and endogenous *MYB80* in the selected lines. (e) Fold changes of expression of *MYB80* direct target genes (*UNDEAD*, *GLOX1* and *GLOX2*) relative to wild type in *At80N32R* transgenic lines. The expression levels of these genes were dramatically reduced in line 6 (sterile) compared to that of in line 12 (75% fertility). (f) Pollen and tapetal development and PCD in the *At80N32R* transgenic line (line 4). Vacuolated tapetal cells and tetrads are identical to wild type at stage 7. Unreleased microspores are observed at stage 9. At stage 11, tapetum shows hypertrophy with reduced cytoplasm. At stage 12, tapetal layer degenerates and collapsed pollen grains remain in the locules. T, tapetum; Msp, microspore; PG, pollen grain. Scale bars = 25 μm in stages 7 and 9, scale bars = 50 μm in stages 11 and 12. (g) At stage 10, the TUNEL signal is first detected in the tapetal cells of both the wild‐type and *At80N32R* anthers (line 4). At stage 11, the TUNEL signal persists in the shrunk tapetal cells of wild‐type anthers and the highly vacuolated tapetal cells of *At80N32R* anthers. Bars = 25 μm. (h) Relative fluorescence intensity of the TUNEL signals in the wild type and *At80N32R* tapetum at anther developmental stages 10 and 11 (n = 3). The relative fluorescent levels of the representative areas were calculated using the background values. Data were generated from the same line as in ‘g’. Error bar represents S.D. (n=3 biological replicates).

Since the N32R motif interacted strongly with the TPL N‐terminus in yeast, the repressive activity of the N32R motif *in planta* was tested. The *N32R* coding sequence was fused with a protein consisting of the *AtMYB80* MYB domain and the conserved 44‐amino‐acid region. The chimeric repressor construct, designated *At80N32R* (Figure 3a), was transformed into wild‐type Arabidopsis. Silique elongation was used to assess the effect of the chimeric repressor on pollen development. We showed previously that the *MYB80‐EAR* constructs interfered with pollen development causing male sterility and silique abortion but did not affect female fertility (Li *et al*., 2007; Phan *et al*., 2012; Xu *et al*., 2014). Consequently, complete silique abortion (failure to elongate and failure to produce any seed) was previously employed as an indicator for complete male sterility and again used in this study. Ninety‐four per cent of the *At80N32R* transgenic lines were male sterile with silique abortion (Figure 3b; Table S1). The *At80N32R* transcript levels were equivalent to or higher than those of endogenous *AtMYB80* (Figure 3c) in the sterile lines analysed. When the cotton homologue of AtMYB80, GhMYB80 was also fused to N32R, a sterile phenotype was also induced in Arabidopsis (Figure 3b and d). Transcripts of two AtMYB80 direct target genes *UNDEAD* and *GLOX1* (Phan *et al*., 2011) were dramatically reduced in a sterile line (Figure 3e). *GLOX2* is a homologue gene to *GLOX1* and both genes showed the same expression patterns (unpublished results). Transcript reduction of *GLOX2* was also obtained in the *At80N32R* lines (Figure 3e).

Tapetum and pollen development in the sterile *At80N32R* lines was examined using light microscopy of anther sections. At stage 7, vacuolation of the *At80N32R* tapetal cells resembled wild type and tetrads of microspores were present in each locule (Figure 3f). At stage 9, the tapetal cells in the *At80N32R* mutant remained highly vacuolated and enlarged. Most of the microspores had not been released. In wild‐type anthers, tapetal degeneration commences at stage 10 and the tapetum becomes a thin layer at stage 11. However, the *At80N32R* tapetal cells remained hypertrophic at stage 11. The cell walls were still visible while the cytoplasmic content was significantly reduced. Vacuolated microspores were present in the locules. At stage 12, the tapetum had completely degenerated. The majority of pollen grains had collapsed and a few aborted grains were attached to the endothecium layer.

TUNEL assays were used to determine the occurrence of PCD in the tapetum and microspores (Figure 3g). As in wild type, prominent TUNEL‐positive signals were first detected in the *At80N32R* tapetal cells at stage 10 and persisted until stage 11. However, the intensity levels of TUNEL signals in the *At80N32R* tapetal nuclei were significantly stronger than those found in wild type (Figures 3h; S3), indicating a more extensive DNA fragmentation had occurred in the *At80N32R* tapetum.

These results indicated that the N32R peptide is an effective EAR motif which converts the MYB80 homologues into dominant repressors which results in strong suppression of MYB80 target genes.

### A novel TOPLESS‐MYB80 chimeric restorer fully rescues the MYB80‐EAR‐induced male sterility

**Figure 4:**
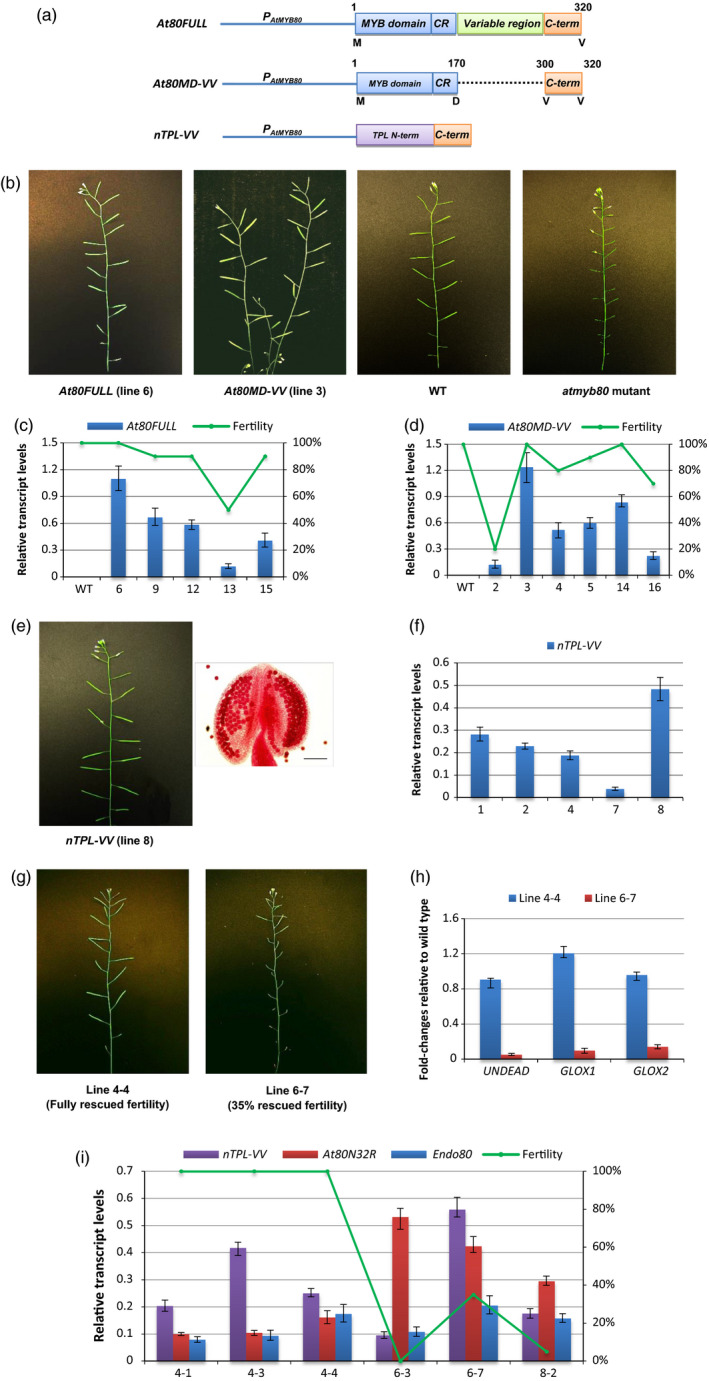
The *nTPL‐VV* chimeric restorer is able to rescue the *MYB80‐EAR* induced male sterility. (a) A schematic diagram (not to scale) of the *At80FULL*,* At80MD‐VV* and *nTPL‐VV* construct. CR, the MYB80 conserved 44‐amino‐acid region; C‐term, the MYB80 conserved final 21‐amino‐acid region. The TPL N‐terminus (188 amino acids) includes the LisH, CTLH and LRD regions. The first and last amino acids for the MYB80 protein sequence or domains are indicated. (b) Male sterility of the *atmyb80* mutant is rescued by either the *At80FULL* or *At80MD‐VV* construct. (c) Plant fertility and relative expression levels of the *At80FULL* transgene in the *atmyb80* mutant. (d) Plant fertility and relative expression levels of the *At80MD‐VV* transgene in the *atmyb80* mutant. (e) An *nTPL‐VV* transgenic line shows normal silique elongation identical to wild type (100% fertility). Alexander's staining of anther shows that all pollen grains exhibit normal morphology. Scale bar = 85 μm. (f) Relative expression levels of the *nTPL‐VV* construct in the selected transgenic plants. All of the transgenic plants appeared fully fertile (i.e. same as wild type). (g) F1 plants possessing both the *At80N32R* repressor and *nTPL‐VV* restorer show phenotypes of restored fertility. (h) Fold changes of expression of *MYB80* direct target genes (*UNDEAD*, *GLOX1* and *GLOX2*) relative to wild type in the *At80N32R/nTPL‐VV* transgenic lines. The expression levels of these genes were restored in line 4‐1 (100% fertility) compared to that of in line 6‐7 (35% fertility). (i) Relative expression levels of the *At80N32R*, *nTPL‐VV* and endogenous *AtMYB80* (*Endo80*) in the selected lines. Expression levels of the transgenes are normalized to *UBQ10*. Error bar represents S.D. (*n* = 3 biological replicates). Lines 4, 6 and 8 of *At80N32R* and lines 2, 7 and 8 of *nTPL‐VV* were used to generate the F1 plants. F1 lines 4‐1, 4‐3 and 4‐4 were generated from the crosses between *At80N32R* parent line 4 with different *nTPL‐VV* parent lines. Likewise, F1 lines 6‐3 and 6‐7 were produced using *At80N32R* line 6 and F1 line 8‐2 using *At80N32R* line 8.

The MYB80 44‐amino‐acid region adjacent to the MYB domain is believed to be required for the binding of the R2R3 MYB domain to *cis*‐elements in the promoters of target genes (Phan *et al*., 2012; Xu *et al*., 2014). Lack of the conserved C‐terminal 18‐ to 21‐amino‐acid sequence abolishes MYB80 function, suggesting MYB80 regulates downstream targets via its conserved C‐terminal region (Li *et al*., 2007). To test whether the variable region is essential for the function of the C‐terminus, a truncated construct (*At80MD‐VV*) was generated by removing the variable region and leaving the final 21 amino acids (from position 300 to 320) intact. Fertility of the *atmyb80* mutant was fully restored by *At80MD‐VV*. A similar result was obtained using the full‐length *AtMYB80* construct (*At80FULL*) (Figure 4a and b; Table S2). The qRT‐PCR results showed At80MD‐VV was able to fully rescue the male sterile phenotype when its transcript levels were similar or equivalent to that of At80FULL (Figure 4c and d). The N‐terminus of MYB80 (MYB domain) is responsible for DNA binding and our results indicate the conserved 21‐amino‐acid C‐terminal sequence engages the transcription complex and hence regulates the expression of MYB80 downstream genes.

To neutralize the repressive activity of the EAR motifs and reactivate the expression of *AtMYB80* downstream genes, a restorer construct (*nTPL‐VV*) was created by combining the LisH–CTLH–LRD region (TPL_1‐188) of the TPL N‐terminus with the 21‐amino‐acid conserved region of the MYB80 C‐terminus. The 21‐amino‐acid sequence was employed as a targeting sequence (TS) to direct the nTPL‐VV protein into the MYB80 transcriptional complex (Figures 1b; 4a). The *nTPL‐VV* construct was first transformed into wild‐type Arabidopsis plants (Col‐0) to determine whether the TPL_1‐188 motif would disrupt pollen development by blocking the EAR motifs present in many transcription repressors expressed in anthers (see Discussion). All 25 transgenic lines obtained were male fertile producing normal pollen and siliques (Figure 4e). Analyses of pollen morphology, flowering time, inflorescence morphology, silique elongation and seed viability revealed no obvious differences from the wild type. Variations in the expression levels of the *nTPL‐VV* transgene did not interfere with pollen development (Figure 4f). These results indicate that the TPL_1‐188 motif did not disrupt the interaction of endogenous TPL/TPR with the EAR motifs present in many anther repressors critical for pollen development. It also shows that the targeting sequence did not interfere with the MYB80 function in the wild‐type plants. Consequently, the *nTPL‐VV* construct was used as a restorer.

F1 plants were obtained by crossing the male sterile plants (female line) possessing the *At80N32R* repressor (lines 4, 6 and 8) with the fertile plants (male line) carrying the *nTPL‐VV* restorer (lines 2, 7 and 8). In total, 14 lines were confirmed to carry both the repressor and restorer transgenes (Figure S4). Plant fertility was fully restored in six lines. The remaining eight lines showed partially fertile phenotypes or were male sterile (Figure 4g; Table S3). Cytoplasmic content of pollen grains in the mature anthers was normal in the fertile lines but completely absent in the sterile lines (Figure S5). The transcript levels of three AtMYB80 direct target genes, namely *UNDEAD*,* GLOX1* and *GLOX2*, were restored to their wild‐type levels in a fully fertile line. The transcript levels remained low in a line which exhibited 35% fertility (Figure 4h).

Transcript levels of *At80N32R* and *nTPL‐VV* in the selected *At80N32R/nTPL‐VV* lines were examined. Sterile or partially fertile phenotypes resulted when the *nTPL‐VV* transcript levels were significantly lower than those of *At80N32R* (lines 6‐3, 8‐2) (Figure 4i). However, higher transcript levels of the *nTPL‐VV* restorer were able to fully restore fertility (lines 4‐1, 4‐3 and 4‐4). The different expression levels of *At80N32R* and *nTPL‐VV* among the F1 lines are likely to be caused by the differences in the transgene copy numbers and the locations of the transgenes in the genome. The phenotype of plant fertility appeared to be determined by the relative expression levels of *At80N32R* and *nTPL‐VV*. The fertile F1 generation lines showed segregated phenotypes in the F2 generation. Genotypic analysis revealed that lines carrying only the *At80N32R* repressor construct reverted to a male sterile phenotype (Table S4; Figure S6), confirming fertility restoration in the F1 generation is a consequence of introducing the *nTPL‐VV* restorer.

### A chimeric *nTPL‐WUSCS* construct induces abnormal phenotypes resembling the *wus‐1* mutant

**Figure 5:**
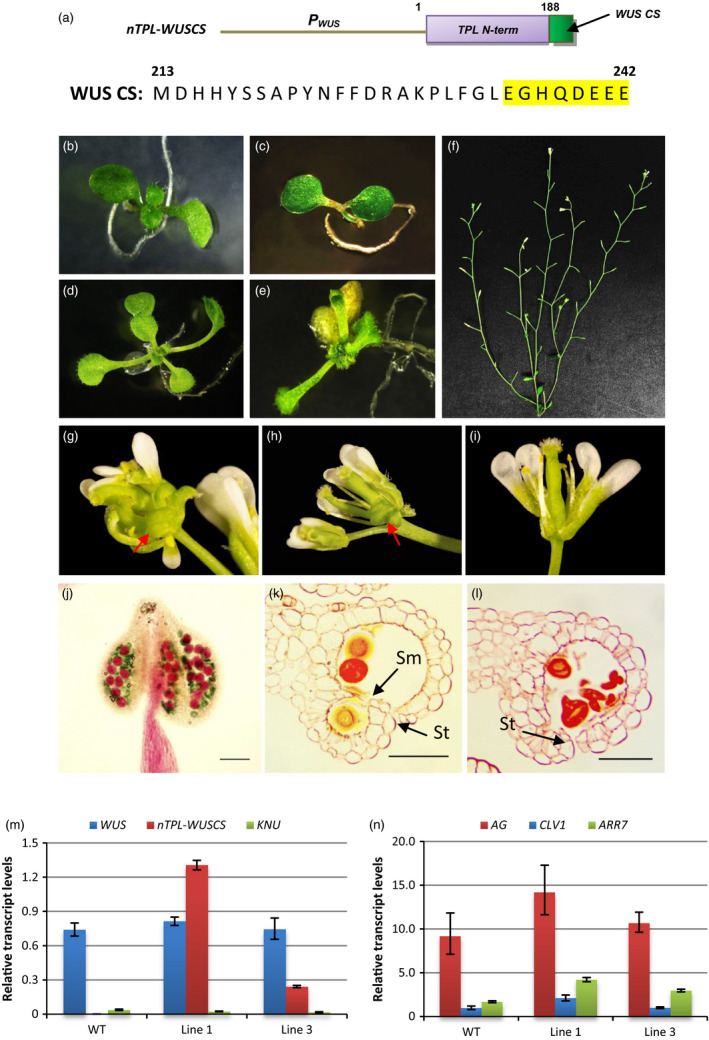
Abnormal vegetative and reproductive phenotypes were induced by the *nTPL‐WUSCS* chimeric construct. (a) A schematic diagram of the *nTPL‐WUSCS* construct. The TPL N‐terminus (N‐term) includes 188 amino acids. The WUS conserved sequence (CS, from the amino acid ‘M’ to ‘E’ at positions 213 and 242, respectively) includes an acidic region (highlighted in yellow). (b–e) Phenotypic analysis of the *nTPL‐WUSCS* young seedlings. A 10‐day‐old *nTPL‐WUSCS* seedling showed defective SAM (c) compared with wild type (b). A 12‐day‐old *nTPL‐WUSCS* seedling exhibited disorganized leaf formation (e) compared with wild type (d). (f) An *nTPL‐WUSCS* plant shows silique abortion. (g–i) Phenotypic analysis of the *nTPL‐WUSCS* flowers. A few pistils were produced in one flower (g and h). Curly and half‐opened styles are indicated by the red arrows. Only four stamens appear in one flower (i). (j) Many pollen grains in the *nTPL‐WUSCS* mature anther developed normally as shown by Alexander's staining. Scale bar = 80 μm. (k and l) Sections showed degeneration of the septum and stomium cells were delayed at stages 13 (k) and 14 (l) in the *nTPL‐WUSCS* anthers. Sm, septum; St, stomium. Scale bars = 50 μm. (m) Relative expression levels of *nTPL‐WUSCS*, endogenous *WUS* and *KNU* in wild type and the selected transgenic lines. Line 1 exhibited a more severe *wus‐1* mutant phenotype than line 3. (n) Relative expression levels of the target genes of *WUS*, including *AG*, *CLV1* and *ARR7* in wild type and the selected transgenic lines. Expression levels of the transgenes are normalized to *UBQ10*. Error bar represents S.D. (*n* = 3 biological replicates).

We next wished to determine whether the technology could be employed to reverse the repressive activity of a known plant repressor, WUSCHEL (WUS). To target and interfere with the repressive activity of the endogenous WUS, a chimeric *nTPL‐WUSCS* construct was made, in which the combined LisH–CTLH–LRD region (TPL_1‐188) was fused with a conserved 30‐amino‐acid sequence of the WUS C‐terminus. The 30‐amino‐acid sequence was employed as the targeting sequence (Figure 5a). The construct was introduced into wild‐type Arabidopsis. In all, 27 of the 60 transgenic young seedlings (lines) displayed abnormal phenotypes. Specifically, defective shoot meristems were initiated in the young *nTPL‐WUSCS* seedlings resulting in disorganized and asymmetric leaf formation (Figure 5c and e). In all, 15 out of the 27 seedlings failed to grow to maturity due to severe apical meristem defect. The surviving 12 lines were examined for phenotypic changes in flowers. These transgenic plants showed severe silique abortion with less than 5% silique elongation (Figure 5f). The disruption of anther dehiscence (Figure 5k and l) contributed to the failure of silique elongation. Multiple carpels were produced from an inflorescence meristem along with a few curly and half‐opened styles (Figure 5g and h) and hence the female fertility may also be affected. In the normally formed flowers, only three to four stamens were present. Few pollen grains were adhesive to the stigma (Figure 5i). Alexander's staining and sections of the mature anthers showed many pollen grains in the locules developed normally although a significant amount lacked cytoplasmic content in the *nTPL‐WUSCS* anthers (Figure 5j). Anther sections showed small and malformed locules. The degeneration of the septum and stomium cells did not occur at anther stages 13 and 14, respectively, resulting in a failure of the anthers to dehisce (Figure 5k and l). The multiple pistils phenotypes were similar to that of the *35S:WUS‐GR* (Kieffer *et al*., 2006) and *LFY:WUS* mutants (Lohmann *et al*., 2001). Otherwise, the phenotypes resembled that of the *wus‐1* mutant (Table S5) (Brand *et al*., 2002; Deyhle *et al*., 2007; Laux *et al*., 1996).

Although the expression level of *nTPL‐WUSCS* was significantly higher in the line exhibiting a more severe phenotype, the transcript levels of endogenous *WUS* in all the lines examined were similar (Figure 5m). *WUS* expression is negatively regulated by *KNUCKLES (KNU)*, which possesses an EAR motif, via a feedback loop (Payne *et al*., 2004; Sun *et al*., 2014). The *KNU* transcript levels were very low compared to those of *WUS* and *nTPL2‐WUSCS* with significant variations between the replicates (Figure 5m). Consequently, the *KNU* expression in *nTPL2‐WUSCS* plants was not significantly different from that of the wild type. These results suggest the abnormal phenotypes which resembled the *wus‐1* mutant were directly induced by the *nTPL‐WUSCS* construct rather than being a consequence of the reduction in *WUS* expression. Moreover, high expression levels of *nTPL‐WUSCS* did not affect the negative regulation of *WUS* by *KNU*. WUS directly represses the expression of *ARABIDOPSIS RESPONSE REGULATOR 7* (*ARR7*) and *CLAVATA1* (*CLV1*) but activates *AGAMOUS* (*AG*) (Busch *et al*., 2010; Ikeda *et al*., 2009; Leibfried *et al*., 2005). Mutating the WUS box results in increased expression levels of *ARR7* in *35S:WUS* lines (Ikeda *et al*., 2009). The transcript levels of *ARR7*,* CLV1* and *AG* were all significantly enhanced in the *nTPL‐WUSCS* line which exhibited more severe phenotypes (Figure 5n). These results indicated that the *nTPL‐WUSCS* chimeric construct had reversed the repressive activity of WUS while retaining its capacity to activate its target genes.

### The TPL_1‐188 domain forms predominantly homomers

**Figure 6:**
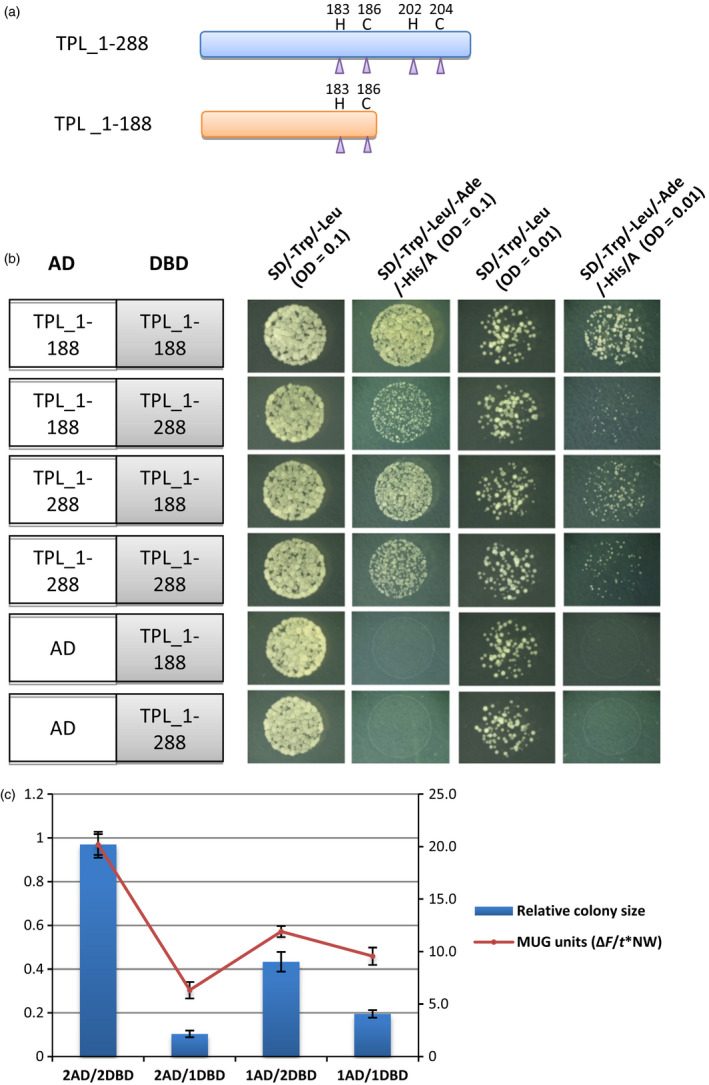
The TPL_1‐188 truncation proteins exhibited strong interactions in yeast cells. (a) A schematic diagram of the TPL_1‐288 and TPL_1‐188 truncation proteins. The purple triangles indicate the positions of Zn^2+^ ions coordinating residues cysteine (C) and histidine (H). (b) Yeast two‐hybrid analysis of interactions between TPL_1‐288 and TPL_1‐188 truncation proteins. Strong interactions occur between the TPL_1‐188‐AD and TPL_1‐188‐BD fusion proteins. A, Aureobasidin A (125 μg/L); AD, activation domain; DBD, DNA‐binding domain. (c) Quantification of the total colony area and fluorescent β‐galactosidase assay of yeast cells possessing the TPL_1‐288 and/or TPL_1‐188 proteins. Yeast cells possessing TPL_1‐188‐AD and TPL_1‐188‐DBD showed significantly higher total colony area and MUG units than those of the TPL_1‐288‐AD/DBD and TPL_1‐188‐DBD/AD, TPL_1‐288‐AD and TPL_1‐288‐DBD proteins. 1AD, TPL_1‐288‐AD; 2AD, TPL_1‐188‐AD; 1DBD, TPL_1‐288‐DBD; 2DBD, TPL_1‐188‐DBD. Error bar represents S.D. (*n* = 3 biological replicates).

The TPL_1‐188 domain might form heteromers with the endogenous TPL protein in the *nTPL‐WUSCS* plants and *At80N32R/nTPL‐VV* plants via their N‐terminal domains (amino acids 1–209). TPL_1‐188 possesses an incomplete N‐terminal domain with the 21 amino acids at the C‐terminus of the domain removed. To examine the homotypic and heterotypic interactions of the TPL_1‐188 and TPL, a yeast two‐hybrid system was used in which the full‐length TPL was replaced with TPL_1‐288 (Figure 6a). The TPL_1‐288 consists of the N‐terminal domain and the proline‐rich sequence (100 amino acids in length). The strengths of the interactions were ascertained using the areas covered by yeast colonies grown in the quadruple dropout medium and by the β‐galactosidase activities (MUG assay). The relative colony areas of the yeast cells possessing TPL_1‐188‐AD/TPL_1‐188‐DBD were significantly larger than those of the yeast cells possessing TPL_1‐288‐AD/TPL_1‐188‐DBD, TPL_1‐288‐AD/TPL_1‐288‐DBD and TPL_1‐188‐AD/TPL_1‐288‐DBD (Figure 6b and c). Similarly, the β‐galactosidase activities expressed as MUG units (see section ‘Materials and Methods’) of the TPL_1‐188/TPL_1‐188 cells were significantly higher than those of the other combinations (Figure 6c). These results indicate that the strongest interaction occurs between the TPL_1‐188 proteins, whereas the interactions to form heterodimers with TPL_1‐288 are much weaker. The TPL_1‐188 possessing the incomplete N‐terminal domain exhibits an enhanced dimerization compared to the TPL_1‐288. Consequently, TPL_1‐188 predominantly forms homomers (dimers/tetramers) rather than heteromers.

## Discussion

### Applications of the CoSRI technology

**Figure 7:**
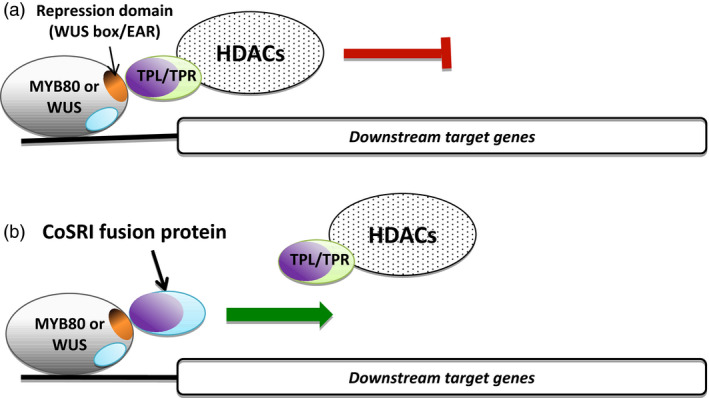
Model of inhibition of the transcriptional repressors using the CoSRI fusion protein. (a) The active repressor (MYB80‐EAR or WUS) recruits the tetramer TPL/TPR co‐repressors and histone deacetylases (HDACs) complex through the repression domains (EAR or WUS box, orange). The expression of downstream genes is supressed. The dimer or tetramer of CoSRI N‐terminus (LisH, CTLH and LRD domains of the TPL/TPR co‐repressors, purple) interacts with the repression domains of the MYB80‐EAR or WUS transcription factors. (b) The CoSRI protein is recruited by the transcriptional machinery through its conserved sequences (blue) of either MYB80 or WUS to compete with the TPL/TPR‐HDAC complex for binding to the repression domains, resulting in reactivation of gene expression.

Two applications of the technology are presented using the CoSRI technology (Figure 7). The repressive activities of an endogenous transcriptional repressor (WUS) and of a specific transcription factor (MYB80) fused with a repression motif (e.g. EAR motif) could be reversed using a chimeric CoSRI protein. The repressor motif‐interacting region of the CoSRI fusion protein binds to the repressor motif of the transcription factor, thereby competing with and displacing the co‐repressor complex. The conserved targeting sequence of the CoSRI protein is recruited by the transcriptional machinery and reactivates the expression of target genes. The mechanism of active transcriptional repression involving the recruitment of co‐repressors by the repressor motifs of transcription factors appears to be shared among eukaryotes. Consequently, the technology should be applicable to manipulating the activity of transcription repressors in crop plants and other eukaryotic cells. The technology can be used to study the cellular functions of repressive motifs and to manipulate the traits regulated by the motifs in crops.

### The N32R EAR motif induces significant transcriptional repression when fused to AtMYB80

The 32R motif is derived from a conserved repression motif found in AtMYB32 (Preston *et al*., 2004). This LxLxL‐type EAR motif showed weak repressive activity when fused to AtMYB80 or GhMYB80. Very few completely male sterile lines were obtained (Xu *et al*., 2014). The modified version of the 32R motif (N32R‐GLDLDLNLELRL) interacted more strongly with the TPL N‐terminus than that of the 32R in the yeast two‐hybrid assay. Hence, the interaction between the N32R peptide and the TPL/TPR co‐repressors *in planta* is stronger than that of 32R resulting in stronger repression of gene activation. When the SRDX EAR motif was fused to the C‐terminal truncated AtMYB80, 60%–75% of the transgenic lines exhibited complete male sterility (Li *et al*., 2007; Phan *et al*., 2012). When the SRDX motif was replaced with the N32R motif in the *AtMYB80* chimeric construct, 94% of the transgenic lines were completely male sterile. Hence, the N32R may be a more efficient repression motif than the SRDX although the transcript levels of the *N32R* and *SRDX* in the transgenic lines need to be compared directly.

Analysis of the *At80N32R* anther cross‐sections revealed that the tapetum layer underwent an abnormal process of development and degeneration. In wild type, the size of tapetal cells decreases from stage 9 until only a thin cell layer remains. However, vacuolation of the *At80N32R* tapetal cell was extended until stage 11 and cells failed to shrink in size. The microspores are released from the tetrad at stage 9 of wild‐type anthers, whereas the microspores were strongly vacuolated and remained together at stage 11 in the *At80N32R* anthers. It is highly likely that expression of *AtMYB80* downstream genes associated with callose dissolution is inhibited by the At80N32R repressor.

Tapetal cell degradation at late anther developmental stages involves apoptosis‐like PCD (Phan *et al*., 2011) during which the cleavage of nuclear DNA can be detected using the TUNEL assay. In the *At80N32R* anthers, prominent TUNEL signals were present in the vacuolated tapetal cells at stages 10 and 11. Relative fluorescence levels of the TUNEL signal were significantly higher in the *At80N32R* tapetum than in wild type, suggesting tapetal PCD was enhanced. Up‐regulation of *UNDEAD* and down‐regulation of *GLOX1* expressions are detected in the *MYB80‐GR* lines after induction by dexamethasone, indicating that AtMYB80 activates *UNDEAD*, but represses *GLOX1* expression (Phan *et al*., 2011). Although At80N32R inhibited *UNDEAD* activation by endogenous *AtMYB80*, it further repressed *GLOX1* expression. Hence, the truncated MYB80 protein produced in the *atmyb80* mutant and the At80N32R repressor influence gene expression differently. For example, *UNDEAD* was down‐regulated 22‐fold in the *atmyb80* mutant (Phan *et al*., 2011), whereas it was barely detectable in the *At80N32R* mutant. *GLOX1* was down‐regulated 19‐fold in the former, whereas it was down‐regulated approximately 100‐fold in the latter. This difference might be responsible for the vacuolation and hypertrophy of tapetal cells from stages 9 to 11 in the *At80N32R* plants, whereas in the *atmyb80* mutant, tapetum and microspores degenerate at stages 10 and 11 (Phan *et al*., 2011). The TUNEL signal first appears at stage 10 in the *At80N32R* plants (stage 9 in the *atmyb80* anthers), but it is stronger than in wild‐type tapetal cells. This implies a higher degree of DNA degradation.

### The MYB80 CoSRI protein acts as an effective restorer to rescue the male sterile MYB80‐EAR lines

The LisH, CTLH and leucine‐rich (LRD) domains at the N‐termini are conserved in the TPL/TPR co‐repressor family (Long *et al*., 2006). The LisH domain is found in many eukaryotic intracellular proteins and may be involved in homodimerization and ATPases/β‐propeller protein interactions in mammalian cells (Emes and Ponting, 2001). The CTLH domain is a predicted α‐helical sequence adjacent to the LisH motif in a co‐repressor homologous family (Emes and Ponting, 2001). None of the individual LisH (36 amino acids), CTLH (59 amino acids) or LRD (96 amino acids) domains interacted with the 12‐amino‐acid N32R motif. However, a strong interaction occurred when the three domains were combined. Ke *et al*. (2015) reported the amino‐terminal domain of the TFL co‐repressor family (TOPLESS domain or TPD) forms an extended disc‐like tetrameric structure. The tetramer is a dimer of dimers. The LxLxL‐type EAR motifs interact with TPL/TPR N‐termini through a shallow groove linked by hydrophobic and positively charged residues in each of the four TPL/TPR monomers. The combined domains constitute the minimal functional region for effectively recruiting various EAR motifs and other repression motifs. Members in the TPL/TPR family may specifically interact with repression motif‐containing regulators via the LisH–CTLH–LRD region.

Deletion of the MYB80 variable region located between the conserved 44‐amino‐acid peptide and the conserved 18‐ to 21‐amino acid C‐terminus did not affect MYB80 function. Fertility restoration (up to 100% fertility) occurred in all *atmyb80* homozygous mutants transformed with *At80MD‐VV*, implying the conserved final 18‐amino‐acid region plays a major role in the MYB80 function. Hence, the short sequence was deployed as a targeting sequence in the nTPL‐VV restorer.

The restorer was first examined in wild‐type plants to ascertain that it does not interfere with pollen development. More than 153 transcription repressors were found to possess the EAR motifs (Causier *et al*., 2012). Using the microarray results of the myb80 mutant versus wild type (Phan *et al*., 2012), we found that at least three genes (IAA13, IAA19 and HAT22) which are differentially expressed in the *myb80* mutant also interact with the TPL/TPR family members (Causier *et al*., 2012). Disruption of several of the EAR‐containing transcription factors which are known to interact with TPL/TPRs interferes with pollen development and male fertility, for example, ARF17 (Yang *et al*., 2013) and MYB32 (Preston *et al*., 2004). The results indicate that the TPL_1‐188 motif of the restorer does not block the EAR motifs of the key anther repressors in wild‐type anthers. The TPL_1‐188 motif may bind to the EAR sequences of the anther repressors with lower affinities than those of the endogenous TPL/TPR protein complexes and hence fail to displace the complexes in the fertile male plants. In the F1 hybrid plants, the MYB80 targeting sequence would guide the restorer to the EAR motif of the repressor MYB80‐EAR (i.e. increasing the EAR‐binding affinity) displacing the TPL/TPR complexes and restoring male fertility (Figure 1b). Although the EAR‐binding affinities of the TPL/TPR complexes could not be compared directly with those of the TPL_1‐188, the EAR (SRDX) binding strength of the longer TPL1‐288 was compared with that of TPL_1‐188 using a yeast two‐hybrid system. The strengths of the interactions were measured using the areas covered by yeast colonies grown in selection medium. The relative colony area of the yeast cells possessing TPL_1‐288‐DBD/SRDX‐AD was larger than that of the yeast cells possessing TPL_1‐188‐DBD/SRDX‐AD (1.56 fold) (Figure S7) indicating stronger interaction between TPL_1‐288 and EAR (SRDX) than that of TPL_1‐188. The EAR‐binding affinities of the endogenous TPL/TPR complexes would presumably be higher than those of TPL_1‐288.

The nTPL‐VV restorer (MYB80 CoSRI) consisting of the TPL LisH–CTLH–LRD region and the AtMYB80 18‐amino‐acid conserved sequence was able to overcome the repressive activity of At80N32R and re‐activate the expression of AtMYB80 target genes. As a result, over 85% of the F1 plants could be restored to fertility and 50% of those lines were completely fertile. The relative expression levels of endogenous *AtMYB8, At80N32R* and *nTPL‐VV* all play a role in determining plant fertility. An approximate 1:1.5 ratio between the At80N32R repressor and the nTPL‐VV restorer is required to fully rescue male sterility. Hence, strong expression of the *At80N32R* repressor is likely to influence the efficiency of fertility restoration.

### Repressive activity of the endogenous WUS protein can be impaired using a WUS CoSRI protein targeting its repression motif

The WUS protein represses the expression of a large number of genes to prevent premature differentiation of stem cells (Busch *et al*., 2010; Schoof *et al*., 2000; Yadav *et al*., 2013). This process of direct transcriptional repression involves the recruitment of the TPL co‐repressor by the WUS repression domains. These domains consist of the WUS box and an EAR motif (Kieffer *et al*., 2006). The repressive activity of WUS was impaired by introducing into wild‐type plants an nTPL‐WUSCS fusion protein (WUS CoSRI) consisting of the N‐terminus of the TPL repressor (LisH–CTLH–LRD) and a 30‐amino‐acid conserved region of WUS. The WUS/WOX proteins interact with the HAIRY MERISTEM (HAM) transcription factors through this 30‐amino‐acid region whose sequence is highly conserved between different species (Zhou *et al*., 2015). As a result, abnormal phenotypes were induced including defective shoot meristems, disorganized leaf formation, abnormal reproductive organ number and dehiscence failure, which resembled that of the *wus‐1* mutant. The expression levels of *ARR7*, one of the WUS direct target genes, are up‐regulated in *nTPL‐WUSCS* mutants. These results indicate that the nTPL‐WUSCS fusion protein specifically blocks the WUS repression domain, thereby weakening the repressive activity of the WUS protein on downstream target genes.

### The TPL_1‐188 fusion proteins regulate the EAR repression motifs via homodimers/tetramers

The N‐terminal domains of TPL/TPR are required for the formation of dimers and tetramers and the interaction with the EAR motifs (Ke *et al*., 2015; Szemenyei *et al*., 2008). Each TPL N‐terminal domain monomer is composed of nine α‐helices followed at the C‐terminus by a loop, which is constrained by a zinc finger structure (Ke *et al*., 2015). The binding of the Zn^2+^ ion is coordinated by four conserved residues, namely two cysteine and two histidine residues with one histidine located in helix 9 and the other three residues in the loop. Helices 1, 2 and 9 mediate the dimerization of the monomer, which forms a new interface required for tetramer formation. The roles of the zinc finger and loop sequence in the dimerization have not been elucidated.

The TPL_1‐188 domain (an incomplete N‐terminal domain) contains only one histidine residue in helix 9 and one cysteine in the loop region with the other two residues removed. The results obtained from the yeast two‐hybrid system show that the removal of the zinc finger and loop sequence enhances TPL_1‐188 homo‐dimerization in comparison with the TPL_1‐288 homo‐dimerization. The TPL_1‐188 predominantly forms homomers rather than heteromers with TPL_1‐288, indicating that the TPL_1‐188 fusion proteins act as homodimers/tetramers in regulating the EAR motifs.

## Experimental procedures

### Plant materials and transformation

*Arabidopsis thaliana* accession Columbia (Col‐0) and an *atmyb80* T‐DNA insertion mutant lines (320C12) (Li *et al*., 2007; Phan *et al*., 2011) were obtained from GABI‐Kat (Max Planck Institute for Plant Breeding Research), the European Arabidopsis Stock Centre. Plants were grown on soil at 22 °C with a 16/8‐h light/night period. Arabidopsis transformation was performed using *Agrobacterium tumefaciens* strain GV3101 by dripping 50 μL of the infiltration medium (Agrobacteria culture suspension at OD_600_ = 0.8, 5% sucrose, 0.03% Silwet) onto each floret. The dripping procedure was repeated once a week for three weeks. Constructs were transformed into the wild‐type or fertile heterozygous *atmyb80* plants. The T_0_ seeds were grown on germination medium containing the appropriate selective antibiotic. Genotypic and phenotypic analysis of the segregating populations was then performed in the T_1_ and T_2_ generations.

### DNA constructs

The coding sequences of the *TPL_1‐288* (864bp), *TPL_1‐188* (564bp), *LisH* (108bp), *CTLH* (177bp), *LRD* (288bp), *LisH*–*CTLH* (276bp) and *CTLH*–*LRD* (465bp) were amplified from anther cDNA by PCR. DNA fragments were then cloned into pGBKT7 vector at the BamHI and EcoRI restriction sites, which were in frame with the *GAL4* DNA‐binding domain. The N32R sequence was designed based on the original 32R motif (LDLNLELRISPP) by replacing the last four amino acids with a leucine (L) and adding another pair of leucine (L) and aspartic acid (D) at the beginning. Glycine (G) was used to separate with other domains. The coding and complementary sequences of *SRDX*,* 32R* (both 42bp) and *N32R* (39bp) were synthesized directly by GeneWorks. DNA fragments were annealed into double strands and cloned into the pGADT7 vector at the EcoRI and BamHI restriction sites, which were in frame with the *GAL4* activation domain. The coding sequences of the WUS conserved sequence (90bp), the full length (*At80FULL*, 963bp) and truncated AtMYB80 or GhMYB80 (both 510bp) were generated by PCR amplification using primers in Table S6. The *TPL* N‐terminus was fused in frame with the *WUS* conserved sequence by overlapping PCR (*nTPL‐WUSCS*), which was then cloned into pCambia 1380 vector (CAMBIA) using the restriction sites PstI and NcoI. A 3.3k *WUS* native promoter which was amplified and cloned into the obtained plasmid using the restriction sites, EcoRI and PstI, was employed to drive the chimeric construct. The N32R sequence was incorporated into the reverse primers to amplify the *At80N32R* and *Gh80N32R* constructs driven by a 1.1k (AtMYB80) and 854bp (GhMYB80) native promoter, respectively. The conserved MYB80 C‐terminus (63bp) was fused in frame with the *MYB80* MYB domain plus 44‐amino‐acid region (*At80MD‐VV*) or *TPL* N‐termini (*nTPL‐VV*) under the control of a 1.1k *AtMYB80* promoter. The DNA fragments of *At80FULL*,* At80N32R*,* Gh80N32R*,* At80MD‐VV* and *nTPL‐VV* were cloned into pENTR/D‐TOPO vector (Life Technologies, Catalog # K240020SP) and then transferred into pGWB501 destination vector. Gene‐specific primers are listed in Table S6.

### RT/qRT‐PCR analysis

Flower bud length was measured and staged according to Peirson *et al*. (1996). Total RNA was extracted from the Arabidopsis young floral buds (anther stages 5–9) using the RNeasy plant kit (Qiagen). The first strand of cDNA was synthesized using SuperScript™ III Reverse transcriptase (Life Technologies, Catalog # 18080–044). The conditions for RT‐PCR amplification of cDNA were as follows: 95 °C for 2 min; 26–28 cycles of 95 °C for 30 s; 58 °C for 20 s and 72 °C for 30 s; one cycle at 72 °C for 5 min. RT‐qPCR was performed using the SensiFAST SYBR & Fluorescein Kit (Bioline, Catalog # BIO‐96020) on the QuantStudio™ 12K Flex Real‐Time PCR system (Applied Biosystems). The PCR conditions were as follows: 95 °C for 2 min; 40 cycles of 95 °C for 20 s; 60 °C for 30 s. Data were analysed using the QuantStudio™ 12K Flex software (Applied Biosystems). Relative gene expression level was calculated using the primer efficiency^(−delta CT)^ method. Fold change was calculated using the primer efficiency^(−delta delta CT)^ method. The Arabidopsis *UBIQUITIN10* (*UBQ10*) gene was used as reference. Three biological replicates were performed for each sample. Gene‐specific primers are listed in Table S6.

### Sectioning of resin‐embedded floral buds

Florets were fixed, embedded and sectioned as described by Li *et al*. (2007).

### Yeast two‐hybrid assay and colony area quantification

Two chimeric constructs encoding the DNA‐binding domain and activation domain fusion proteins were transformed into the Y2HGold Yeast Strain (Clontech, Catalog # 630498) which possesses four reporter genes (*AUR1‐C*,* ADE2*,* HIS3* and *MEL1*). The fresh grown yeast colonies were evenly dotted onto the double (−Trp/−Leu) or quadruple dropout (−Trp/−Leu/−Ade/−His) medium mixed with X‐α‐Gal (40 mg/L) and Aureobasidin A (125 μg/L). Yeast cells were incubated at 30 °C for 3 days. To quantify colony area, the number of yeast cells was controlled by normalizing to OD600 = 0.1 or 0.01 after growing overnight in the double dropout medium. The normalized yeast culture (20 μL) was spotted onto double or quadruple dropout medium mixed Aureobasidin A (125 μg/L) in triplicate. Yeast cells were incubated at 30 °C for 2 days. Image J software (http://rsb.info.nih.gov/ij/) was employed to calculate the total colony area (by square centimetres). Three repeats were performed for each sample. Relative colony size was calculated by comparing the total colony area of samples on quadruple dropout medium to that on double dropout medium.

### Fluorescent α‐galactosidase assay of yeast cells

The concentrated yeast whole‐cell extracts (WCE) were prepared following the protocol described by Visweswaraiah *et al*. (2011). An aliquot of 50 μL of yeast WCE with the breaking buffer (30 mm HEPES, pH 7.4, 50 mm KCl, 10% Glycerol and protease inhibitor) was transferred into a 96‐well cell culture plate (Cellstar, Catalog # 655180). An aliquot of 20 μL of 1 mg mL^−1^ 4‐Methylumbelliferyl‐β‐D‐galactopyranoside (MUG) was added into each well, and samples were incubated at room temperature for 15 min. The reaction was stopped by adding 30 μL of 1 m Na_2_CO_3_. The generated fluorescence was then measured in a Synergy 2 Multi‐Mode Microplate Reader (BioTek) with an excitation filter at 360 nm and an emission filter at 460 nm. The arbitrary MUG units (F_360/460_/t ∙ A_595_, where F_360/460_, t and A_595_ are the sample fluorescence at the end of the reaction, time of reaction in minutes and absorbance of the cell suspension, respectively) of α‐galactosidase activity described by Vidal‐Aroca *et al*. (2006) were calculated for each sample. Data were normalized using a cell‐free sample as a blank reference.

### TUNEL assay and TUNEL signal quantification

The whole Arabidopsis inflorescences were incubated in the fixing solution (4% paraformaldehyde in PBS, 0.1% Triton X‐100 and 0.1% Tween‐20) for 15 min at room temperature under vacuum. This was repeated for 3 or 4 times with slow pulling and release of vacuum. Fresh fixing solution was used to incubate samples overnight at 4 °C. Samples were then dehydrated in a graded ethanol series, and cleared in the solutions of ethanol/Histo‐Clear (National Diagnostics, Catalog # HS‐200) at the percentage of 75%/25%, 50%/50% and 25%/75% for 1 h each and twice in 100% Histo‐Clear for 1 h each time. The individual floral buds were infiltrated with the melted paraplast (Sigma‐Aldrich, Catalog # P3683) at 60 °C for 8 h. Samples were oriented in foil boats and hardened overnight. Sections of 6 μm were cut using a rotary microtome (Jung AG Heidelberg) and attached to silane‐coated slides. TUNEL assay was performed using the DeadEnd™ Fluorometric TUNEL System (Promega, Catalog # G3250) according to the manufacturer's instructions. Slides were counterstained with 1 μg/mL of propidium iodide and mounted with SlowFade^®^ Gold Antifade Mountant (Life Technologies, Catalog # S36936). Samples were examined under a laser scanning spectral confocal microscope (Leica, model TCS SP2) using a spectrum of excitation at 488 nm and emission between 509 nm to view the green fluorescence of fluorescein, and a spectrum of excitation at 543 nm and emission over 620 nm to view the red fluorescence of propidium iodide (Life Technologies, Catalog # P1304MP). The Image J software was employed to generate the merged images and measure the fluorescence intensity. To determine the relative fluorescent level, a small circle of fixed size (52 pixels) was drawn on the TUNEL‐positive nuclei and the adjacent TUNEL‐negative tapetal areas. Three repeats were performed for each sample at the same anther developmental stage. The relative fluorescent level of the representative areas was calculated as the subtraction of the average measurements of background values from the average measurements of integrated densities for each sample.

## Supporting information


**Figure S1** Expression analyses of *TOPLESS* in Arabidopsis anther using semi‐quantitative RT‐PCR.
**Figure S2** Yeast two‐hybrid analysis of interactions between EAR motifs and TPL N‐termini with X‐α‐Gal substrate.
**Figure S3** Selection and quantification of TUNEL signals in the tapetal cells.
**Figure S4** Genotypic analysis of the *At80N32R* and *nTPL‐VV* transgenes in the F1 generation lines.
**Figure S5** Alexander's staining of the F1 *At80N32R/nTPL‐VV* anthers.
**Figure S6** Genotypic analysis of the *At80N32R* and *nTPL‐VV* transgenes in the F2 generation lines.
**Figure S7** Quantification of the total colony area of yeast cells possessing SRDX EAR and TPL_1‐288 or TPL_1‐188 protein.
**Table S1** Plant fertility and number of the *At80N32R* and *Gh80N32R* transgenic lines.
**Table S2** Plant fertility and number of the *myb80* homozygous mutants possessing either *At80FULL* or *At80MD‐VV* construct.
**Table S3** Plant fertility and number of the F1 generation lines possessing both the *At80N32R* and *nTPL‐VV* transgenes.
**Table S4** Plant fertility and genotypic analysis of the *At80N32R/nTPL‐VV* F2 generation lines.
**Table S5** A comparison of phenotypes between the *wus‐1*,* 35S:WUS‐GR, LFY:WUS* and *nTPL‐WUSCS* mutants.
**Table S6** Primer sequences used in this article. Nucleotide sequences are in the 5′ to 3′ order.
