# Supplementary material for: Regulation of gene expression by manipulating transcriptional repressor activity using a novel CoSRI technology
Source: Plant Biotechnol J. 2017 Mar 10;15(7):879–93. doi: 10.1111/pbi.12683 (PMC5466438; doi:10.1111/pbi.12683)
Supplement: Supplementary file 1 — Figure S1 Expression analyses of TOPLESS in Arabidopsis anther using semi‐quantitative RT‐PCR. Figure S2 Yeast two‐hybrid analysis of interactions between EAR motifs and TPL N‐termini with X‐α‐Gal substrate. Figure S3 Selection and quantification of TUNEL signals in the tapetal cells. Figure S4 Genotypic analysis of the At80N32R and nTPL‐VV transgenes in the F1 generation lines. Figure S5 Alexander's staining of the F1 At80N32R/nTPL‐VV anthers. Figure S6 Genotypic analysis of the At80N32R and nTPL‐VV transgenes in the F2 generation lines. Figure S7 Quantification of the total colony area of yeast cells possessing SRDX EAR and TPL_1‐288 or TPL_1‐188 protein. Table S1 Plant fertility and number of the At80N32R and Gh80N32R transgenic lines. Table S2 Plant fertility and number of the myb80 homozygous mutants possessing either At80FULL or At80MD‐VV construct. Table S3 Plant fertility and number of the F1 generation lines possessing both the At80N32R and nTPL‐VV transgenes. Table S4 Plant fertility and genotypic analysis of the At80N32R/nTPL‐VV F2 generation lines. Table S5 A comparison of phenotypes between the wus‐1, 35S:WUS‐GR, LFY:WUS and nTPL‐WUSCS mutants. Table S6 Primer sequences used in this article. Nucleotide sequences are in the 5′ to 3′ order. [file PBI-15-879-s001.docx]

Regulation of gene expression by manipulating transcriptional repressor activity using a novel CoSRI technology

Yue Xu, Song Feng Li and Roger W. Parish

Department of Animal, Plant and Soil Sciences, La Trobe University, AgriBio - Centre for AgriBioscience, Melbourne, Victoria 3083, Australia

**Supplemental Materials**

**
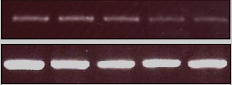
**

**β*-tubulin* (26 cycles)**

***TOPLESS* (28 cycles)**

**Figure S1** Expression analyses of *TOPLESS* in Arabidopsis anther using semi-quantitative RT-PCR. *TOPLESS* was strongly expressed in anthers at stages 5 to 9. Five biological replicates were performed. The β*-tubulin* was employed as the reference gene.

**a b**


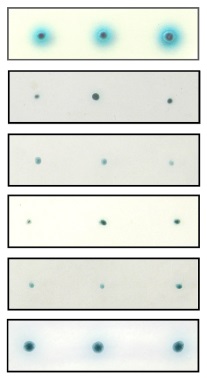


**DBD N32R-AD**

**TPL_1-188 (LisH-CTLH-LRD)**

**CTLH (34-92)**

**LisH (1-36)**

**LRD (93-188)**

**LisH-CTLH (1-92)**

**CTLH-LRD (34-188)**

**DBD**

**AD**


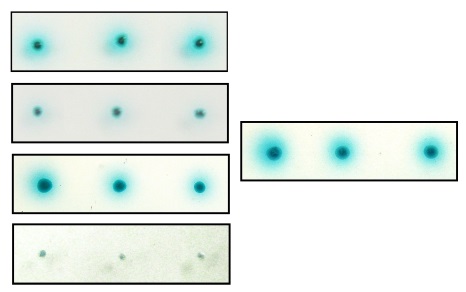


**SRDX**

**32R**

**N32R**

**Negative Control**

**Positive Control**

**TPL _1-288**

**Figure S2** Yeast two-hybrid analysis of interactions between EAR motifs and TPL N-termini with X-α-Gal substrate. (**a**) Yeast colonies possessing the TPL _1-288-DBD and 32R-AD fusion proteins show relatively weak colours, while yeast colonies possessing the TPL _1-288-DBD and SRDX-AD or N32R-AD fusion proteins exhibit strong blue colour surrounding the colonies. (**b**) Yeast colonies possessing the LisH-CTLH-LRD-DBD and N32R-AD fusion proteins show strong surrounding colours. Blue colour reflects β-galactosidase activity. Numbers indicate amino-acid residues of TPL N-terminus. AD, activation domain; DBD, DNA-binding domain. Positive control shows the interaction between pVA3-1 and pDT1-1. The negative control indicates the absence of interaction between the empty AD vector and TPL _1-288-DBD.

**Wild type**

***At80N32R***


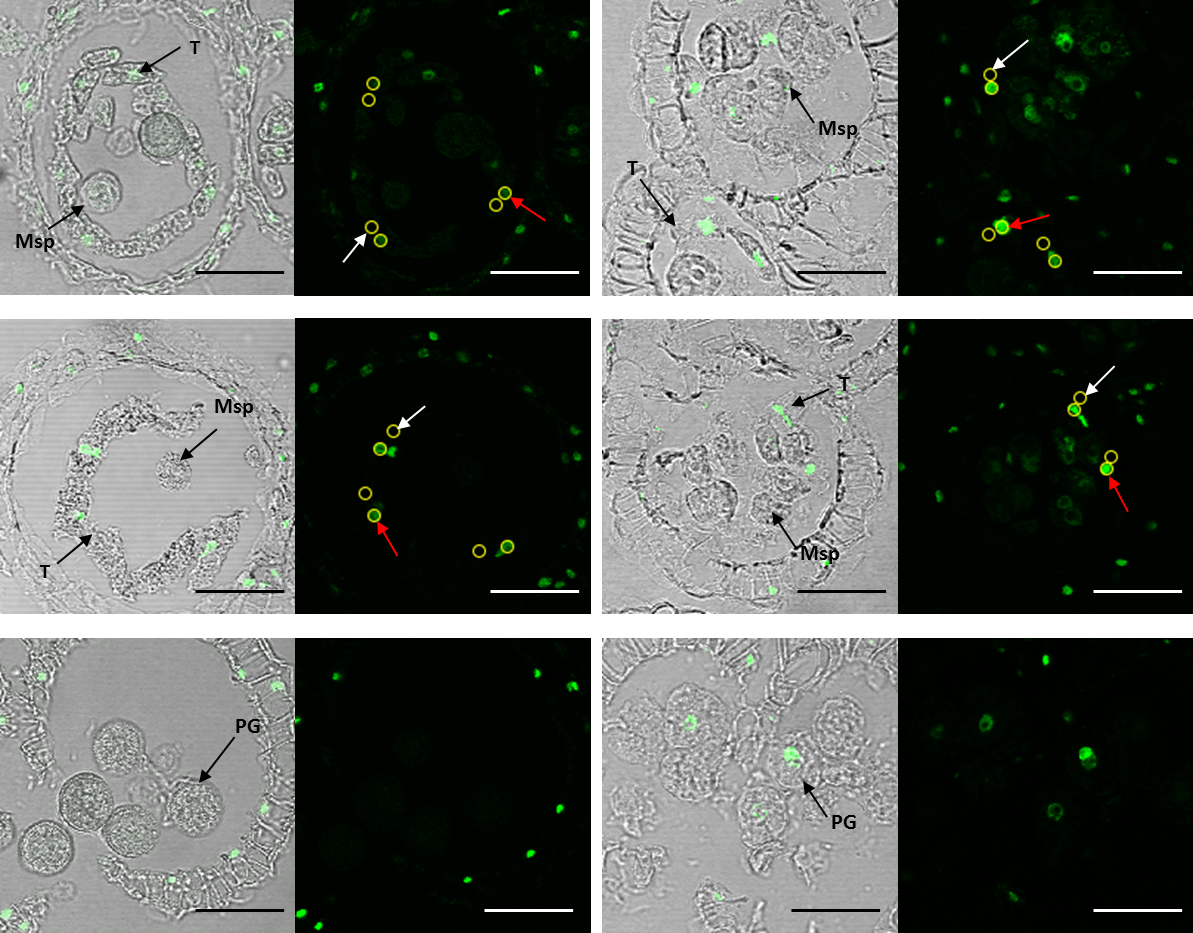


**Stage 10**

**Stage 11**

**Stage 13**

**a b**

**c d**

**e f**

**Figure S3** Selection and quantification of TUNEL signals in the tapetal cells. Left panels are the overlay of DIC images with the fluorescence of TUNEL signals. Right panels are the fluorescence of TUNEL signals only. The TUNEL-positive (red arrows) and background signals (white arrows) of the wild-type (**a** and **c**) and *AtN32R* (**b** and **d**) tapetum at stages 10 and 11 were selected. The florescence intensity of each selected area was measured using ImageJ software. The degradation of the tapetum completed at stage 12. The TUNEL signal is visible in the collapsed *AtN32R* pollen grains (**f**) but not in the wild type (**e**) at stage 13. T, taptem; Msp, microspore; PG, pollen grain. Bars = 25 mm.


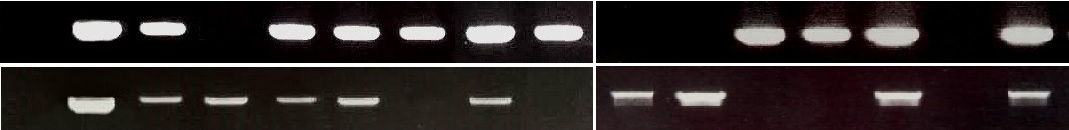


***At80N32*R**

***nTPL-VV***

**1 2 3 4 5 6 7 8 9 10 11 12 13 14 15 16**

**Figure S4** Genotypic analysis of the *At80N32R* and *nTPL-VV* transgenes in the F1 generation lines. Gel electrophoresis results show the presence of *At80N32R* and/or *nTPL-VV* transgenes in the F1 offspring of *At80N32R* repressor line 4 crossed with *nTPL-VV* restorer lines. Lane 1, negative control; lane 2 positive control; lane 3 to 16, 4-1 to 4-14.


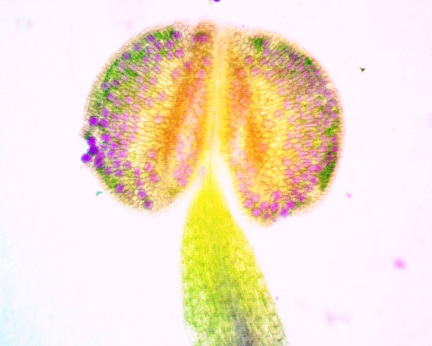

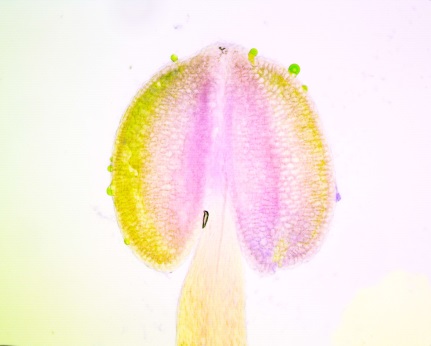

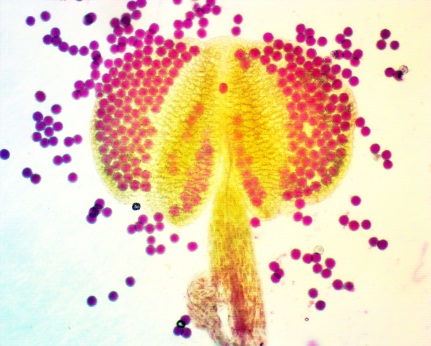


**a**

**b**

**c**

**Figure S5** Alexander’s staining of the F1 *At80N32R/nTPL-VV* anthers. (**a)** Fully fertile *At80N32R/nTPL-VV* anther shows that pollen grains developed normally. (**b)** Partially fertile *At80N32R/nTPL-VV* anther shows reduced number of pollen grains compared to the fully fertile anthers. Some pollen is collapsed and lacking viable cytoplasm (green staining). (**c)** Completely sterile *At80N32R/nTPL-VV* anther shows the absence of well-developed pollen grains in the locules.

**
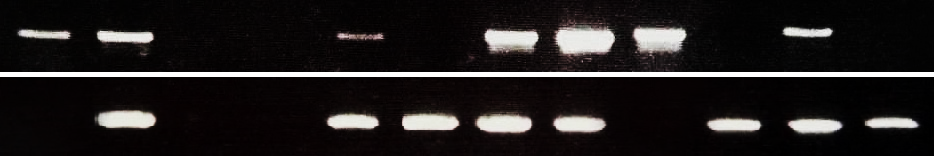
**

***At80N32*R**

***nTPL-VV***

**1 2 3 4 5 6 7 8 9 10 11 12**

**Figure S6** Genotypic analysis of the *At80N32R* and *nTPL-VV* transgenes in the F2 generation lines. Gel electrophoresis results show the presence of *At80N32R* and/or *nTPL-VV* transgenes in the twelve F2 generation lines (lanes 1 to 12) of the *At80N32R/nTPL-VV* selfing offspring.

**Figure S7** Quantification of the total colony area of yeast cells possessing SRDX EAR and TPL_1-288 or TPL_1-188 protein. Yeast cells possessing TPL_1-288-DBD and SRDX-AD showed higher total colony area than those of the TPL_1-188-DBD and SRDX-AD proteins. 1AD, DBD, DNA binding domain; AD, activation domain. Error bar represents S.D. (n=3 biological replicates).

**Table S1** Plant fertility and number of the *At80N32R* and *Gh80N32R* transgenic lines.

| Plant fertility (%) | Number of transgenic *At80N32R* lines | Number of transgenic *Gh80N32R* lines |
| --- | --- | --- |
| 0 | 66 | 19 |
| 25 – 50 | 2 | 33 |
| 50 –75 | 1 | 18 |
| 100 | 1 | 15 |

**Table S2** Plant fertility and number of the *myb80* homozygous mutants possessing either *At80FULL* or *At80MD-VV* construct.

| Plant fertility (%) | Number of *atmyb80/At80FULL* lines | Number of *atmyb80/At80MD-VV* lines |
| --- | --- | --- |
| 0 – 15 | 0 | 3 |
| 15 – 50 | 2 | 4 |
| 50 – 90 | 4 | 7 |
| 100 | 9 | 5 |

**Table S3** Plant fertility and number of the F1 generation lines possessing both the *At80N32R* and *nTPL-VV* transgenes.

| Plant fertility (%) | Number of *At80N32R/nTPL-VV* transgenic lines |
| --- | --- |
| 0 | 2 |
| 5-10 | 3 |
| 30-50 | 3 |
| 100 | 6 |

**Table S4** Plant fertility and genotypic analysis of the *At80N32R/nTPL-VV* F2 generation lines.

| Plant fertility (%) | Number of lines | Genotype |
| --- | --- | --- |
| 0 | 10 | *At80N32R* |
| 20-40 | 19 | *At80N32R/nTPL-VV* |
| 100 | 14 | *nTPL-VV* |
| 100 | 18 | *At80N32R/nTPL-VV* |

**Table S5** A comparison of phenotypes between the *wus-1*, *35S:WUS-GR, LFY:WUS* and *nTPL-WUSCS* mutants.

|  | *wus-1* | *35S:WUS-GR or LFY:WUS* | *nTPL-WUSCS* |
| --- | --- | --- | --- |
| Shoot Meristem | 7-day-old seedlings lack a shoot apical meristem (Laux et al., 1996; Brand et al., 2002). | NA | 10-day-old seedlings lack a shoot apical meristem. |
| Reproductive organs | Flowers lack most of the central organs, stamens and carpels. Only a single or up to three stamens are formed (Laux et al., 1996). | Multiple flowers with curly styles initiate from a large fasciated inflorescence meristem in the *35S:WUS-GR* mutant (Kieffer et al., 2006).  Floral organ number is increased. Extra gynoecia surround the normal central gynoecium in the *LFY:WUS* mutant. (Lohmann et al., 2001). | The number of stamens is reduced. Three to four stamens are produced in one flower. Multiple carpels with curly and half-opened styles are generated from an inflorescence meristem. |
| Vegetative development | Disorganized bunches of cauline leaves (Laux et al., 1996). | NA | Disorganized and asymmetric leaf formation. |
| Anther and pollen morphology | Anthers have smaller or malformed locules. Septum and stomium fail to degeneration at later stages. Anthers do not dehisce and release pollen grains. Pollen grains are viable (Deyhle et al., 2007). | NA | Locules are small and malformed. The septum and stomium cells fail to degenerate at the later stages result in a failure to dehisce. Few pollen grains are adhesive to the stigma. Many pollen grains in the locules appear to be normal. |
| Fertility | Sterile | NA | Less than 5% fertility |

**Table S6** Primer sequences used in this article. Nucleotide sequences are in the 5’ to 3’ order.

| Name | Nucleotide sequence |
| --- | --- |
| Vector construction |  |
| SRDX-F | CAGTGAATTCGGTTTAGATTTGGACCTTGAACTCAGGCTAGGATTTGCGTAAGGATCCATCG |
| SRDX-R | CGATGGATCCTTACGCAAATCCTAGCCTGAGTTCAAGGTCCAAATCTAAACCGAATTCACTG |
| 32R-F | CAGTGAATTCGGTTTGGACTTAAATCTGGAGCTAAGAATAAGTCCTCCTTAAGGATCCATCG |
| 32R-R | CGATGGATCCTTAAGGAGGACTTATTCTTAGCTCCAGATTTAAGTCCAAACCGAATACACTG |
| N32R-F | CAGTGAATTCGGTTTAGATTTGGACTTAAATCTGGAGCTAAGACTATAAGGATCCATCG |
| N32R-R | CGATGGATCCTTATAGTCTTAGCTCCAGATTTAAGTCCAAATCTAAACCGAATTCACTG |
| TOP-F | GAGGCCGAATTCATGTCTTCTCTTAGTAGAGAGCTCG |
| nTPL1-R | GTCGACGGATCCTTAATTTTTACAAAGCTGGTGTTGCCAATT |
| nTPL2-R | GTCGACGGATCCTTATGGAGGAGTTCTCGGGTGCTTC |
| LisH-R | GTCGACGGATCCTTAGAAGAAAAACCCAGATTCTTGTTC |
| CTLH-F | GAGGCCGAATTCGGGTTTTTCTTCAATATGAAGTAT |
| CTLH-R | GTCGACGGATCCTTACTTGGGACGATCATGCTTATCCAA |
| LRD-F | GAGGCCGAATTCGCTGTGGATATACTAGTGAAGGA |
| pWUS-F | GCATCGAATTCCTGGAGGGACGGGTTTTTGAATCAAC |
| pWUS-R | GACATCTGCAGGTGTGTTTGATTCGACTTTTGTTCAC |
| TPL-WUS F | CACACCTGCAGATGTCTTCTCTTAGTAGAGAGCTCG |
| TPL-WUS R1 | GAAGAAGTTGTAAGGTGCAGATGAGTAATGATGATCCATCGGGCCATTTTTACAAAGCTGGTGTTGCCAATT |
| TPL-WUS R2 | GATCGCCATGGCTATTCTTCTTCTTCTTGATGACCTTCTAGACCAAACAGAGGCTTTGCTCTATCGAAGAAGTTGTAAGGTGCAGATGAG |
| pAt80-F | CACCGGCAAGGAGCTTCTATGGCCAA |
| At80-N32R | TTATAGTCTTAGCTCCAGATTTAAGTCCAAATCTAAACCGTCAACACGTTTCTTGGTGAGC |
| pGh80-F | CACCAAAACCCATTTTAACTGTAATAAATC |
| Gh80-N32R | TTATAGTCTTAGCTCCAGATTTAAGTCCAAATCTAAACCATCGATACGTTTCTTAGTTAGCAG |
| At80FULL-R | CCAAATAAAAATCAAACCATATG |
| At80MDVV-R | TCAAACCATATGATTGATGAGATCATCAGCTTGTAAATCCCACAAGACATTATCCACATTAAACACCGGGCCGTCAACACGTTTCTTGGTGAGCAA |
| pAt80-R | TTCTTCTTTCTTTCTTTCTAG |
| pAt80-TPL F | CTAGAAAGAAAGAAAGAAGAAATGTCTTCTCTTAGTAGAGAGCTCG |
| TPL-VV R | TCAAACCATATGATTGATGAGATCATCAGCTTGTAAATCCCACAAGACATTATCCACATTAAACACCGGGCCATTTTTACAAAGCTGGTGTTGCCAATT |
| RT-PCR/qPCR |  |
| WUS-F | CATGCAAGCTCAGGTACTGAATGTGG |
| WUS-R | GACCTTCTAGACCAAACAGAGGC |
| qTPL-F | GGGGACACCAAGTCTGCAAGA |
| TPL-WUS R | GGTGCAGATGAGTAATGATGATCC |
| KNU-F | CGTCCTCGCTAACTCTCCAC |
| KNU-R | ACGGATGAAACGGATCGTAG |
| AG-F | TTGGAAGGCAGATTAGAGAGAAG |
| AG-R | CTGGAGAGCGGTTTGGTCTTGGC |
| CLV1-F | GGTGCTGCTTCTGAGTGTATGTC |
| CLV1-R | CTTCCCAGCTATTAACTCCAAC |
| ARR7-F | CAGCATTCAGAGAAGTACCAGTAG |
| ARR7-R | GCATTCCTCAGCTTCATTCCTC |
| qAtMYB80-F | TTCTCGCATCTAATGGCAGAG |
| AtN32R-R | CCAGATTTAAGTCCAAATCTAAACC |
| GhN32R-R | CATGGCTGAGATAGCCACTAC |
| Endo80-F | GAGGAGGAGGAGAGAAGGAA |
| Endo80-R | CATCAGCTTGTAAATCCCAC |
| UND-F | CACTCTACATATCCCTCTTGTGC |
| UND-R | CTGCTACCCGTATCCATGTGA |
| Glox1-F | GTTGGGCTCACTCTGTCCTCG |
| Glox1-R | GTGGATAGGTACCTTGCCGTG |
| Glox2-F | GGTCGATTGTTGGGCTCATTCG |
| Glox2-R | CATCTCTTCGCGGCCAATGCC |
| qAt80FULL-R | CGCCCACCCTTTCAAACCATATG |
| qAt80MDVV-R | ATCCACATTAAACACCGGGCCGTCAA |
| UBQ-F | TCCGGATCAGCAGAGGCTTA |
| UBQ-R | TCAGAACTCTCCACCTCAAG |
